# Bioturbation by mammals and fire interact to alter ecosystem-level nutrient dynamics in longleaf pine forests

**DOI:** 10.1371/journal.pone.0201137

**Published:** 2018-08-22

**Authors:** Kenneth L. Clark, Lyn C. Branch, Jennifer Farrington

**Affiliations:** 1 USDA Forest Service, Northern Research Station, New Lisbon, New Jersey, United States of America; 2 Department of Wildlife Ecology and Conservation, University of Florida, Gainesville, Florida, United States of America; Pacific Northwest National Laboratory, UNITED STATES

## Abstract

Activities of ecosystem engineers can interact with other disturbances to modulate rates of key processes such as productivity and nutrient cycling. Bioturbation, movement of soil by organisms, is a widespread form of ecosystem engineering in terrestrial ecosystems. We propose that bioturbation by southeastern pocket gophers (*Geomys pinetis*), an abundant but declining ecosystem engineer in longleaf pine (*Pinus palustris* Mill.) forests, accelerates nutrient dynamics of the forest floor by burying litter and then reduces litter consumption and nitrogen (N) volatilization losses in the presence of fire. We evaluated our hypothesis by measuring how litter burial alters decomposition and N and phosphorus (P) turnover of longleaf pine and turkey oak (*Quercus laevis* Walt.) litter over four years, and then simulated interactive ecosystem-level effects of litter burial and low-intensity fires on N and P dynamics of the litter layer. In the field, mass loss was over two times greater and N and P were released much more rapidly from litter buried beneath mounds than on the surface of the forest floor. At a measured rate of mound formation covering 2.3 ± 0.6% of the forest floor per year, litter mass and N and P content of the forest floor simulated over an eight-year period were approximately 11% less than amounts in areas without pocket gopher mounds. In contrast to unburied litter, litter beneath mounds is protected from consumption during fires, and as fire interval increased, consumption rates decreased because mounds cover more years of accumulated litter. Our research indicates that bioturbation and burial of litter by pocket gophers accelerates turnover of N and P on the forest floor, and in the presence of fire, conserves N in this ecosystem where productivity is known to be nutrient limited.

## Introduction

Ecosystem engineers that modify rates of key processes can have disproportionately large effects on ecosystem structure, productivity and nutrient cycles [1–4]. Where activities of ecosystem engineers interact with and mitigate impacts of other disturbance processes (e.g., flooding or fire), these activities may represent important, but often overlooked, mechanisms contributing to ecosystem functioning [4–7]. Further, recognition and incorporation of the interactions between ecosystem engineers and disturbance into ecosystem restoration efforts potentially enhances their success. Bioturbation, the movement of soil by organisms, is a widespread form of ecosystem engineering in terrestrial ecosystems, ranging from alpine meadows to temperate and tropical forests, which has the potential to interact with a broad set of ecological processes (e.g., [6,8,9]). Through burrowing, foraging, and other behaviors that move soil and mix surface and subsurface soil, animals alter hydrology and soil properties [10] and change the spatial distribution of soil organic matter and nutrients [8,9,11]. Another frequently occurring outcome of bioturbation is burial of plant litter and waste products [12,13]. Studies of fossorial mammals in alpine meadows and semiarid scrub have demonstrated that burial of litter by burrow soil can alter rates of decomposition and nutrient dynamics by changing the microenvironment of buried litter (i.e., temperature and moisture) [13–15]. Research in fire-prone ecosystems of Australia has emphasized the potential of bioturbation to influence wildland fire behavior because burial of the litter layer reduces the amount and alters the spatial configuration of fine fuel available for consumption during fires [7,12]. An important next step in understanding ecosystem-level impacts of bioturbation is to link these two sets of outcomes as bioturbation occurs in fire-adapted ecosystems around the world [7,11,16]. In addition, many vertebrate ecosystem engineers involved in bioturbation, such as fossorial mammals, large reptiles, and large ground birds are declining and the ecological functions of these species are being lost before they are fully understood [7,17–20].

**Fig 1:**
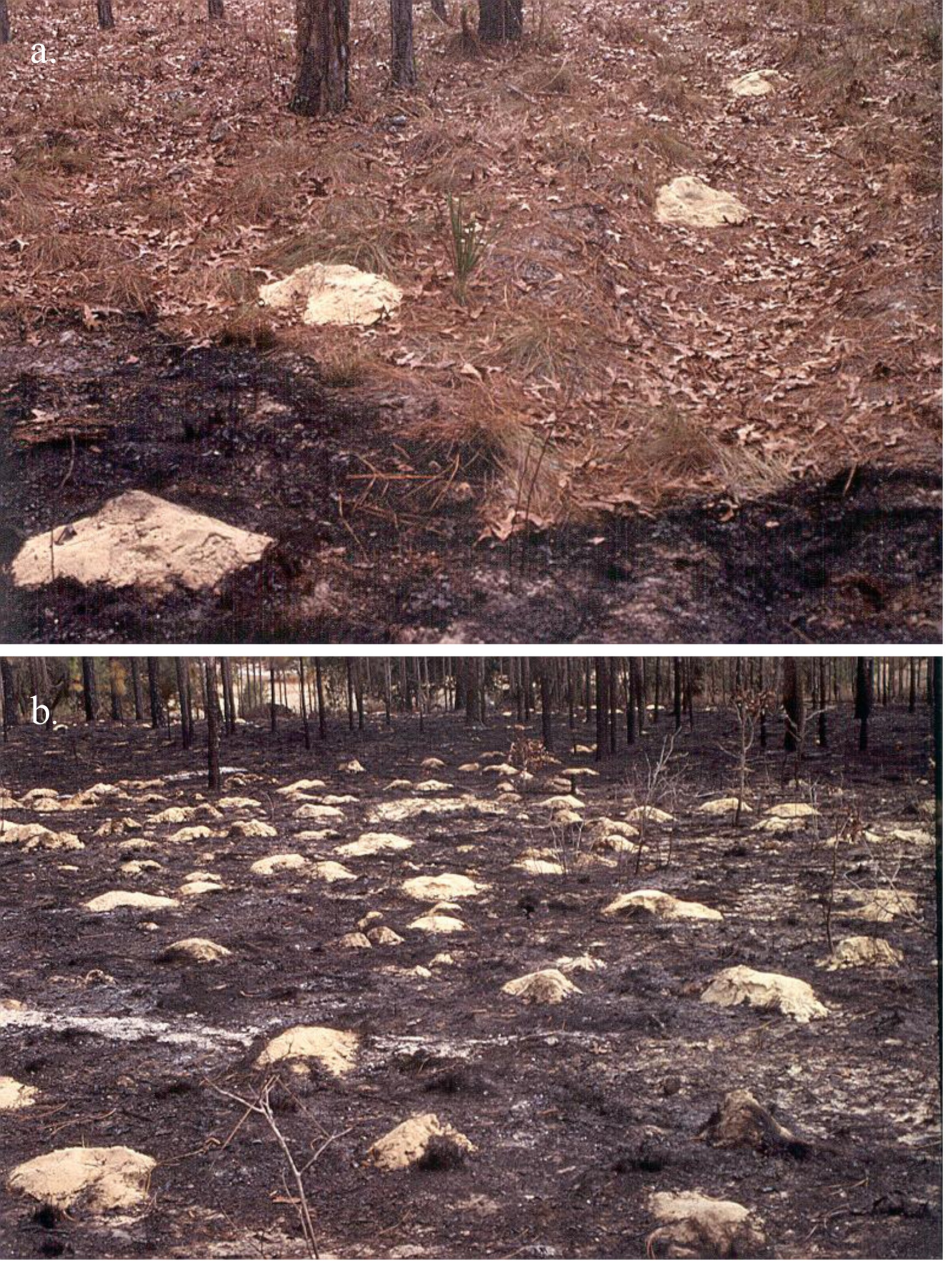
Pocket gopher mounds in a longleaf pine forest at the Ordway-Swisher biological station. (a) Pocket gopher mounds covering longleaf pine and turkey oak litter near the edge of a recent burn. In the burned section, a large portion of the litter was consumed by the fire, but litter under pocket gopher mounds was protected from consumption. (b) Pocket gopher mounds have a clumped distribution, often resulting in locally high densities.

Our study focuses on bioturbation in longleaf pine forest (*Pinus palustris* Mill.), an oligotrophic, fire-adapted ecosystem in the southeastern USA [21–23]. In this system a suite of bioturbators, including southeastern pocket gophers (*Geomys pinetus*), gopher tortoises (*Gopherus polyphemus*), and burrowing beetles (e.g., *Peltotrupes youngi*) excavate large quantities of soil and form surface mounds that bury litter on the forest floor [24,25]. Pocket gophers typically are responsible for the greatest amount of animal-generated soil disturbance in longleaf pine forests [26], with up to 2,500 recently formed mounds occurring per ha [24] covering 2.2 to 4% [24], or greater, of the forest floor (Fig 1). Longleaf pine forests historically were characterized by high-frequency, low-intensity fires, which maintained an open understory structure and promoted a very high diversity of herbaceous species [21,23]. As humans have encroached on this system, naturally ignited fires have been largely replaced by prescribed burning [21,22,27]. Because goals and constraints for land management differ across the region, fire intervals vary widely (e.g., 1–10 years, or more) [23,28]. Frequent low-intensity burns (e.g., 1–5 year intervals) can result in the maintenance of desirable stand structure resembling historic conditions [23,29,30], but also deplete nitrogen (N) on the forest floor and in vegetation by volatilization, particulate transport, and erosion [31–34].

Considerable efforts are aimed at restoration of the longleaf pine ecosystem, which now occupies less than 5% of its original range [35–38]. Restoration activities have included silvicultural treatments targeting undesirable hardwood species, perennial shrubs, and invasive species, enrichment planting of longleaf pine and other desirable species, and the fine-tuning of prescribed fire frequency, intensity and seasonality to promote the high diversity of understory species [21,23,27,30,36–38]. Ecosystem engineers, such as bioturbators, increasingly are cited for their important role in ecosystem restoration in other fire adapted ecosystems [7,39]. However, we lack a complete understanding of how bioturbation potentially enhances ecosystem functioning of longleaf pine forests. Additionally, ecosystem engineers in longleaf pine forests may be in decline and have become locally extinct over large areas, thus are of conservation concern. For example, southeastern pocket gophers and gopher tortoises are listed in statewide conservation action plans as high priority across their range because of their biological vulnerability, population declines, and roles as keystone species [40–44].

We hypothesized that through mound building and interactions of these mounds with fires, bioturbators have contrasting effects on litter layer mass and nutrient dynamics, alternatively accelerating and decelerating nutrient turnover and loss, respectively. We examined our hypothesis by focusing on the burrowing activities of the southeastern pocket gopher. We predicted that pocket gophers accelerate decomposition and nutrient release by mounding soil over litter, but that this activity also decreases the amount of litter available for consumption during fires, resulting in a deceleration of N volatilization and phosphorus (P) pyro-mineralization. To examine these predictions, we quantified how burial of the litter layer by pocket gophers affects decomposition and N and P dynamics of litter, and then simulated how litter burial by pocket gophers and frequent low-intensity fires interact to alter litter consumption and nutrient turnover. More specifically, we conducted field censuses to quantify the rate of new pocket gopher mound formation, measured litterfall over a three–year period to estimate mass, N and P inputs to the forest floor, and used a litterbag study over a four–year period to estimate decomposition rates and N and P dynamics of longleaf pine needles and turkey oak (*Quercus laevis* Walt.) foliage on the forest floor and buried beneath pocket gopher mounds. We then integrated this information with previously published estimates of litter layer consumption during low-intensity prescribed fires in a simulation model to evaluate 1) ecosystem-scale effects of pocket gopher mound formation on litter layer dynamics, and 2) to explore the interactive effects of mound formation rate and fire return interval on N and P dynamics of the litter layer.

## Materials and methods

### Site description

Research was conducted at the Ordway-Swisher Biological Station in north-central Florida, USA (29.6893, -81.9934), a core NEON site for the Southeast domain (See www.ordway-swisher.ufl.edu). The biological station comprises approximately 4300 ha of upland longleaf pine forest, mesic hammocks, wet prairie, and ponds. The climate is humid and warm temperate, with average monthly air temperatures of 12.4°C in January and 27.6°C in July (National Climatic Data Center, 1981–2010). Average annual precipitation is 1242 mm, with greater amounts occurring during the summer months. Soils are deep, excessively drained yellow sands belonging to the Candler (hyperthermic, uncoated lamellic quartzipsamments) and Apopka series (loamy, siliceous, subactive, hyperthermic grossarenic paleudults) and are characterized by very low organic matter and nutrient content [45].

The vegetation in upland forests of the Ordway-Swisher Biological Station is dominated by longleaf pine (*Pinus palustris* Mill.) and turkey oak (*Quercus laevis* Walt.) in the overstory, and wire grass (*Aristida stricta* Michx.), gopher apple (*Licania michauxii* Prance), and shiny blueberry (*Vaccinium myrsinites* Lamark) in the understory. A high diversity of other forb and prairie grass species also occur in the understory. Southeastern pocket gophers (*Geomys pinetis*), as well as other mound forming species (e.g., burrowing beetles and gopher tortoises), are common in upland forests throughout the biological station. The fire return frequency in longleaf pine stands over the past 30 years generally has been two to five years, but some areas have reached more than 10 years between fires [46] (S. Coates pers. comm.).

### Pocket gopher mound censuses

Twelve 0.5–ha plots were established at random locations in each of three 1–km^2^ upland areas of uneven aged longleaf pine forest. All pocket gopher mounds in these plots were marked with pin flags in late fall of the first year of the study. We then counted and marked all new pocket gopher mounds that were formed with uniquely colored pin flags at three to six–month intervals over a two–year period. The area of forest floor covered by a subset of recently formed mounds (n = 150) was estimated by measuring the widest and narrowest distance of each mound, and the average area of forest floor covered by each mound was calculated as an ellipse. Pocket gopher mound census data are available at [https://datadryad.org/resource/Pocket Gopher Mound Census Clark et al.xlsx; DOI to be added].

### Litterfall collection

Three 1–m^2^ litterfall traps were placed at random locations in three of the 12 half hectare plots in each of the three upland areas for a total of 27 traps. Litter was collected approximately bi-monthly when present, separated into pine, oak, other foliage, wood, and reproductive and miscellaneous material, dried at 70°C for at least 72 hours, and then weighed. Litter samples were then pooled by plot (n = 3 traps) for each collection period and subsamples were ground for carbon (C), nitrogen (N) and phosphorus (P) analyses. Litterfall and C, N and P data are available at: [https://datadryad.org/resource/Mass Nitrogen and Phosphorus in Litterfall Clark et al.xlsx; DOI to be added].

### Litter decomposition

Litter decomposition was estimated using litterbags placed on the surface of the forest floor and under pocket gopher mounds. Fresh, recently fallen litter consisting of longleaf pine needles or turkey oak leaves was collected from the forest floor in and around plots during the peak of litter production from late November to early January and returned to the laboratory for processing. Approximately 5.0–g equivalent dry weight of air-dried longleaf pine needles (pine), turkey oak foliage (oak), or 2.5–g of each (mixed) were weighed and placed in each litterbag (10 x 20 cm constructed from 1–mm mesh size nylon screen). Initial mass and N and P content of litter were estimated from air-dried samples that were weighed, dried at 70°C for at least 72 hours, weighed again, and then ground for analyses. Litterbags were placed beneath pocket gopher mounds and on the surface of the forest floor adjacent to all 12 plots in each of the three 1–km^2^ upland areas. Recently-formed pocket gopher mounds were located near the perimeter of each plot, and a litterbag was carefully inserted under the center of each mound at the top of the litter layer with a metal spatula to minimize disturbance to buried plants and the forest floor beneath mounds. For each buried litterbag, we placed a second litterbag containing the same type of litter on the surface of the forest floor at one meter in a random direction, and litterbag pairs were marked with labeled pin flags located between each pair. Six replicate pairs of all three types of litterbags (pine, oak, mixed) were placed adjacent to each plot (36 plots, 1296 litterbags in total). One set of litterbags (i.e., six bags with the three litter types from the surface and buried locations) was harvested from each plot after 6, 12, 18, 24, 36, and 48 months. By 36 and 48 months, some litterbags were lost, and damaged litterbags were excluded from further analyses. In the laboratory, roots and any extraneous material on the outside of the litterbags were removed, and sand was then brushed carefully from litter bag contents. Any fine roots within pine or oak litter samples were separated, and litter and roots were dried at 70°C for at least 48 hours and weighed when dry. Litter decomposition and N and P content data are available at: [https://datadryad.org/resource/Mass Nitrogen and Phosphorus in Litterbags Clark et al.xlsx; DOI to be added].

### Chemical analyses

Ash free mass of subsamples of pooled litterfall and all harvested litterbag samples was estimated by loss on ignition of dry samples in a muffle furnace at 550°C. Carbon content of litter and litterbags was estimated on a subset of samples (n = 15 initial litterbag samples and n = 5 each of randomly selected litterbags containing pine or oak litter harvested at each sampling period) using a CNS analyzer (Carlo Erba, Milan, Italy). Total N and P in pooled litterfall samples, initial litterbag samples, and all harvested litterbags were estimated using modified Kjeldahl procedure: 0.25 g of dry plant tissue was digested with a sulfuric acid/hydrogen peroxide/potassium sulphate/copper sulphate mixture using a block digester at 375° C. Digests were analyzed for N and P using a Technicon Autoanalyzer at the Forage Evaluation Support Laboratory, University of Florida.

### Data analyses

Litterfall mass was multiplied by the N and P content of the appropriate litter type for each collection period, and values were summed to calculate annual mass and N and P flux to the forest floor. We calculated a negative exponential decay constant (k) for the rate of mass loss for each litter type in litterbags in each 0.5–ha plot using SigmaPlot (Version 12.5, Systat Software, Inc., San Jose, CA, USA). Following Olson [47], the form of the model is y = e^-k t^, where y is the fractional mass remaining at time t in years. k values were compared among litter types (pine, oak, mixed) and locations (forest floor, buried) with linear mixed models constructed with the lmer function in R package Ime4 (see citations [48,49]). The R code used for all statistical analyses is presented in S1 Appendix. We also used linear mixed models in R to evaluate how burial of litter by pocket gophers affected N and P dynamics after 24 and 48 months during the decomposition process. Mixed buried litterbags were omitted from P analyses at 48 months because of a small sample size resulting from missing and damaged litterbags that were excluded from further analyses. We then used linear mixed models to evaluate the effect of pine versus oak litter on ingrowth of fine root biomass into litterbags. We limited our analyses to buried litterbags because most litterbags on the forest floor had no root ingrowth. Comparisons among litter types and location were made with Tukey´s method that adjusts *p* values for multiple comparisons using the lsmeans package in R [50]. Prior to analyses, all data were evaluated to meet statistical assumptions. Values of k for each type of litter and P content of decomposing litter at 24 months were not normally distributed, and these data were log-transformed before analyses. The relationship between percent mass remaining and N and P concentrations in litterbag samples also was examined with regression analyses using SigmaPlot.

### Simulation of litter layer dynamics

We used rates of new mound formation estimated from the pocket gopher mound censuses, average annual litterfall flux values measured over three years, and decay constants and net changes in N and P contents of litter during decomposition derived from the litterbag study to develop a set of spreadsheet models in Excel in the Microsoft Office 2013 suite (Microsoft Corp., Redmond, WA, USA) to simulate mass loss and N and P dynamics of the litter layer. Details of the simulations, including model inputs, calculations, outputs, and simulated scenarios and their assumptions are provided in S2 Appendix. In summary, decomposition and N and P dynamics of annual “cohorts” of four types of litter (surface pine, surface oak, buried pine, buried oak) were simulated separately, and values for all appropriate cohorts were summed to calculate remaining mass and N and P content of the litter layer each year. We simulated five scenarios; 1) litter layer dynamics in the absence of pocket gophers (no disturbance simulations), 2) litter layer dynamics of a single mound over a 10–year time period (single mound simulation), 3) effects of litter burial by pocket gophers at five annual rates of new mound formation (mound density simulations), 4) effects of low-intensity prescribed fires conducted at periodic intervals using previously published values for forest floor consumption in longleaf pine forests (prescribed fire simulations), and 5) interactive effects of litter burial and low-intensity fire on mass, N and P dynamics of the litter layer (mound density and fire simulations). Results from all simulations are available at: [https://datadryad.org/resource/Pocket Gopher Mound and Fire Simulations Clark et al.xlsx; DOI to be added].

#### No disturbance simulations

For the no disturbance simulations, we initially estimated when Phase 1 decomposition products, defined as 20% of initial mass remaining [51,52], stabilized on the forest floor using mean annual pine and oak foliar litterfall flux and decay constants calculated from litterbags. Nitrogen and P contents in decomposing litter were calculated as a function of remaining litter mass. Simulations were then extended beyond Phase 1 to estimate when all organic matter and amounts of N and P derived from pine and oak foliage stabilized on the forest floor.

#### Single mound simulation

We used a single mound simulation to evaluate how burial of the litter layer and subsequent accumulation of litter on top of an individual mound altered litter layer mass, N and P dynamics. The mound was assumed to be average size and buried an amount of litter equivalent to “steady state” amounts predicted by the no disturbance simulation. We also assumed that mean annual litterfall amounts accumulated on top of the mound every year following formation. Decay constants derived from buried litterbags were used to calculate mass loss from buried pine and oak litter, and those from surface litterbags were used to calculate mass loss from litter that accumulated on top of the mound following formation. Nitrogen and P content in decomposing litter was calculated as the appropriate function of remaining litter mass in each annual cohort. Annual cohorts of the four litter types (buried pine, buried oak, surface pine, surface oak) were then summed to calculate mass and N and P content of the litter layer each year.

#### Mound density simulations

We evaluated how the density of newly formed pocket gopher mounds altered litter layer mass and N and P dynamics by simulating five annual rates of new mound formation, covering no (0% yr^-1^), low (1% yr^-1^), medium-low (2.3% yr^-1^), medium-high (5% yr^-1^), and high (10% yr^-1^) area of the forest floor per year. Pocket gophers have a clumped distribution, and rates of mound formation and cover of the forest floor by mounds can vary widely across the landscape. A value of 2.3% yr^-1^ represents the percentage of forest floor covered by mounds at the maximum annual rate of mound formation measured in a 0.5–ha plot in this study (712 mounds ha^-1^ yr^-1^). Other studies in longleaf pine forest have reported annual rates of mound formation of 442–916 mounds ha^-1^ yr^-1^ [24,26]. The percentage of the forest floor covered by mounds in parts of the plots where mounds were aggregated likely exceeded 10% yr^-1^ (Fig 1B). Single mound simulations were weighted by the proportion of forest floor covered by new mounds per year, and no disturbance simulations were used for areas without mounds. Similar to previous simulations, N and P content in each annual cohort of the four types of litter were calculated as a function of remaining litter mass. Annual cohorts of each litter type were then summed to calculate mass and N and P content of the litter layer every year.

#### Prescribed fire simulations

We simulated litter layer consumption during low-intensity fires based on published values for longleaf pine forests [53,54] and mixed southern pine forests [55]. Reid et al. [53] reported an average consumption of fine fuels in the litter layer of 53.3 ± 14.5% for dormant and growing prescribed burns (n = 45) in longleaf pine stands. This value falls near the middle of the range of consumption values (11 to 100%) reported by Prichard et al. [55] during 60 fires in North Florida in mixed southern pine forest that contained some longleaf pine stands. We used an estimated value of 50% consumption during each fire and simulated effects of prescribed fires at 3, 5 and 10–year intervals on litter layer mass and N and P content, based on typical fire return intervals employed at Ordway Swisher Biological Station. We assumed that all annual cohorts of the litter layer with > 20% remaining were partially consumed during fires, and that N was volatilized and P was pyro-mineralized in the same proportions as litter was consumed. Following each burn, we assumed that remaining litter that was not consumed decomposed at the same rate as unburned surface litter. We also assumed that no change to annual litterfall amounts, composition or nutrient content occurred following each burn.

#### Mound density and fire simulations

To evaluate how the density of pocket gopher mounds reduces litter layer consumption during prescribed fires, we simulated the interactive effects of pocket gopher mound density and prescribed burn intervals on litter layer mass and N and P dynamics of the litter layer. Five densities of new mounds covering 0%, 1%, 2.3%, 5% and 10% of the forest floor per year and prescribed fires at three, five and ten year intervals were simulated. Annual litter cohorts were simulated as in the mound density simulations for newly formed mounds, and as in the prescribed burn simulations for areas without pocket gopher mounds. For older mounds that burned, we assumed that average annual amounts of litterfall accumulated on top of mounds through time, and decomposition and N and P dynamics were modeled as in the no disturbance simulation until prescribed burns occurred. We also assumed that reduced litter amounts on newer mounds had no effect on consumption during prescribed burns, thus 50% of initial litter amounts were consumed in all locations. All litter cohorts with > 20% initial mass remaining were summed for each year, and values are presented as percent reduction in litter layer consumption, N volatilization and P pyro-mineralization as a function of simulated rates of annual mound formation. Details of all simulations are in S2 Appendix.

## Results

### Pocket gopher mound censuses

Recently-formed pocket gopher mounds were encountered in all 36 0.5–ha plots at some time during the study. Average mound size was 0.32 ± 0.01 m^2^ (mean ± 1 SE, range 0.15–0.62 m^2^, n = 150). Mounds were highly clustered, and local densities of new mounds could cover > 10% of the forest floor (Fig 1B). The greatest number of new mounds was encountered during fall and winter censuses, when maximum rate of new mound formation over a three-month period was 241 mounds in a 0.5–ha plot. On an annual basis, the maximum number of new mounds encountered in a 0.5–ha plot was 356 mounds, equivalent to 2.3 ± 0.6% of the forest floor covered per year. The average number of mounds encountered across all 0.5–ha plots in each 1–km^2^ forest area was 15 ± 23, 39 ± 61 and 77 ± 104 mounds per year.

### Litterfall

**Fig 2:**
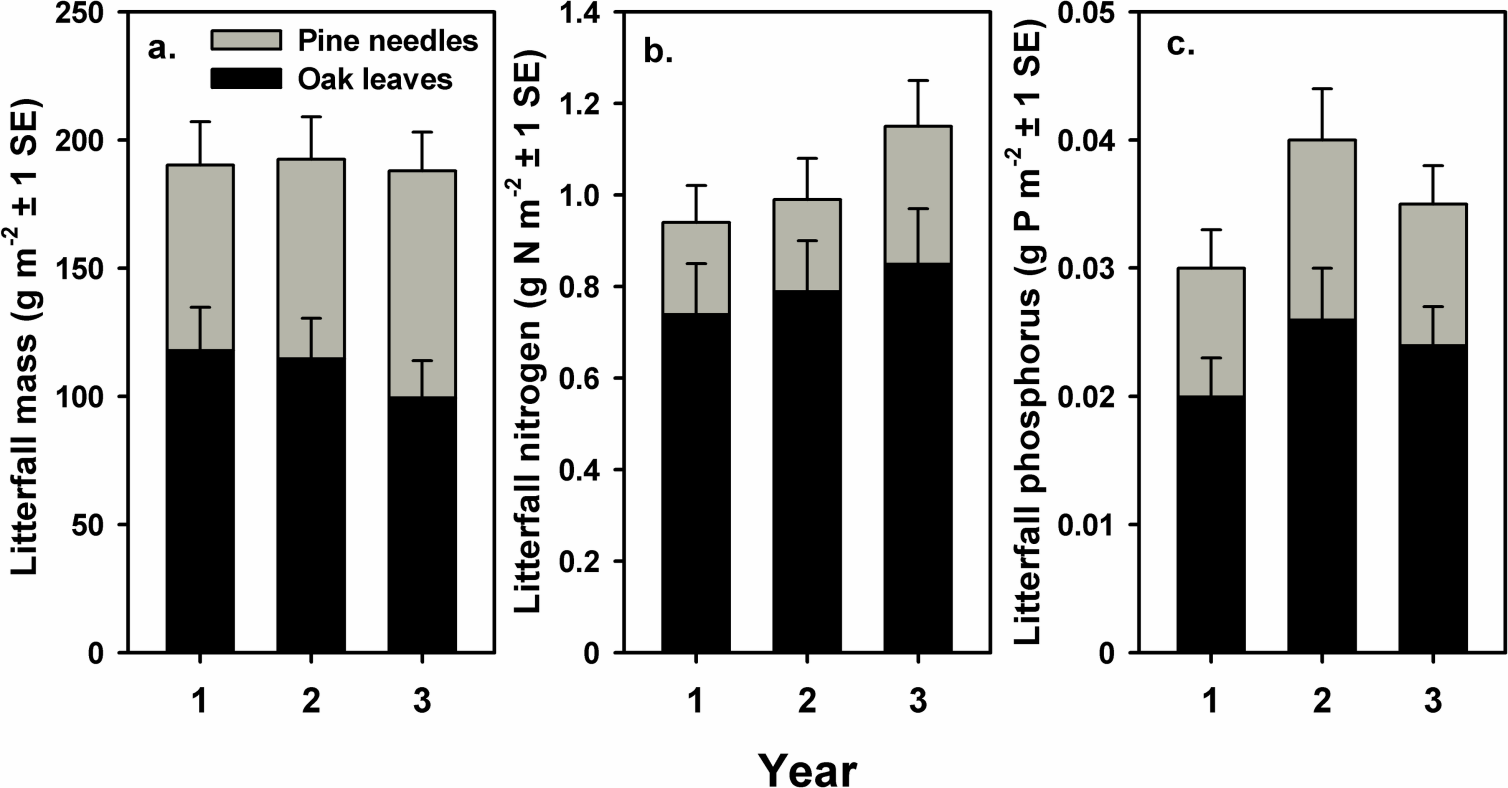
Annual fine litterfall flux derived from canopy foliage in longleaf pine forest at the Ordway-Swisher biological station. Values are mean g m^-2^ yr^-1^ ± 1 SE from 27 litterfall traps; a) mass, b) nitrogen, and c) phosphorus.

Average fine litterfall totaled 245 ± 23 g m^-2^ yr^-1^ (mean ± 1 SE) and was composed of 31% pine needles, 45% turkey oak leaves, and 23% woody, reproductive and miscellaneous material (Fig 2; S1 Table). Annual N and P flux in litterfall derived from canopy foliage averaged 1.03 g N m^-2^ yr^-1^ and 0.035 g P m^-2^ yr^-1^ (Fig 2B and 2C). Turkey oak accounted for 78% and 67% of the annual N and P flux in litterfall derived from canopy foliage, respectively.

### Litter decomposition

**Fig 3:**
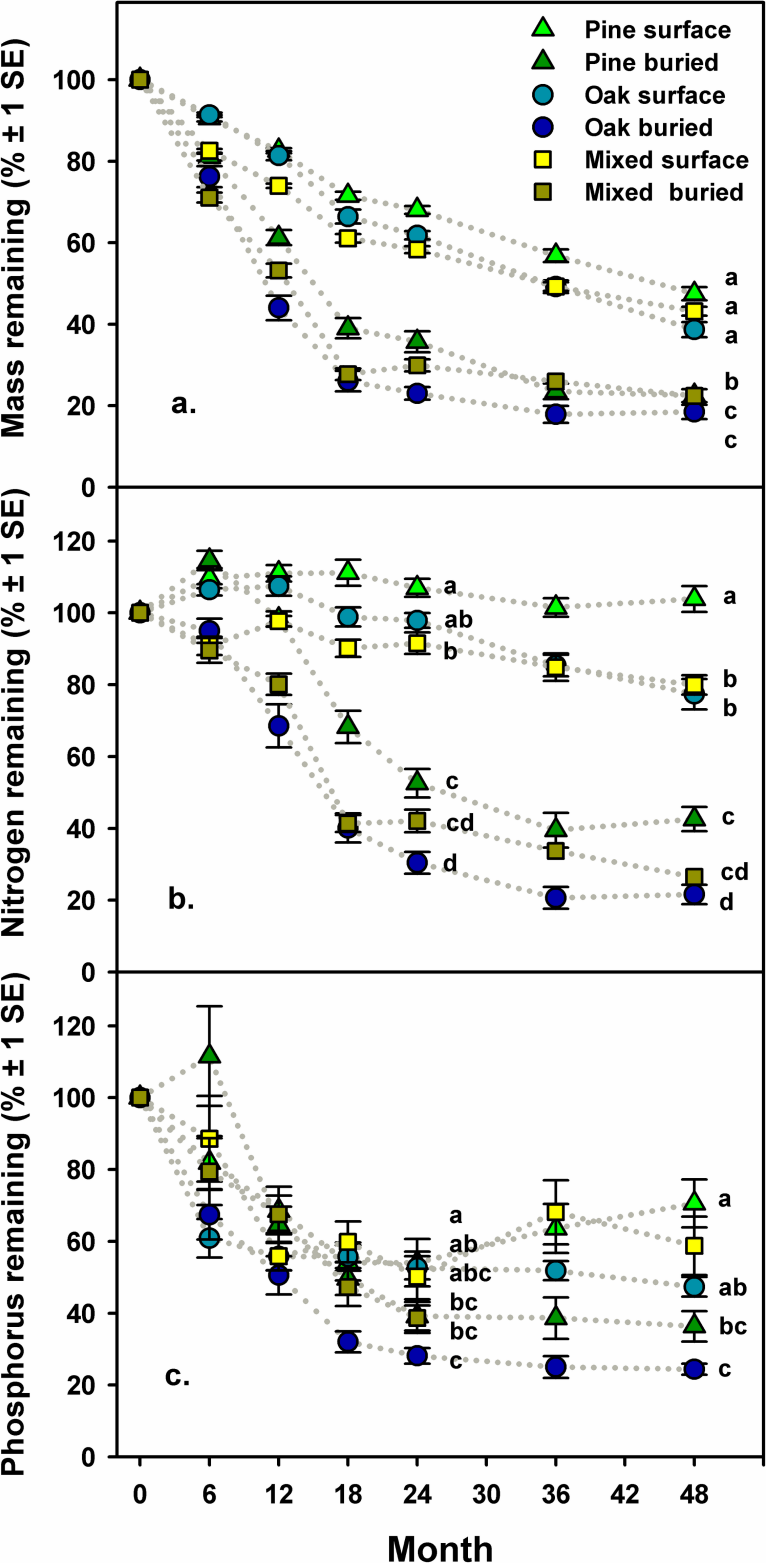
**Decomposition and nitrogen and phosphorous dynamics of longleaf pine needle litter, turkey oak leaf litter, and mixed pine and oak litter in litterbags on the surface of the forest floor and buried beneath pocket gopher mounds over a four year period;** a) percent of the initial mass remaining, b) percent of the initial nitrogen content remaining, and c) percent of the initial phosphorous content remaining. Values are means ± 1 SE. Litter types and location that are significantly different (*p* < 0.05) have different letters. Significance levels for percent mass remaining were calculated based on models for decomposition coefficients, k, calculated for sets of litterbags in each 0.5 ha plot. Complete statistics are in Table 2.

**Fig 4:**
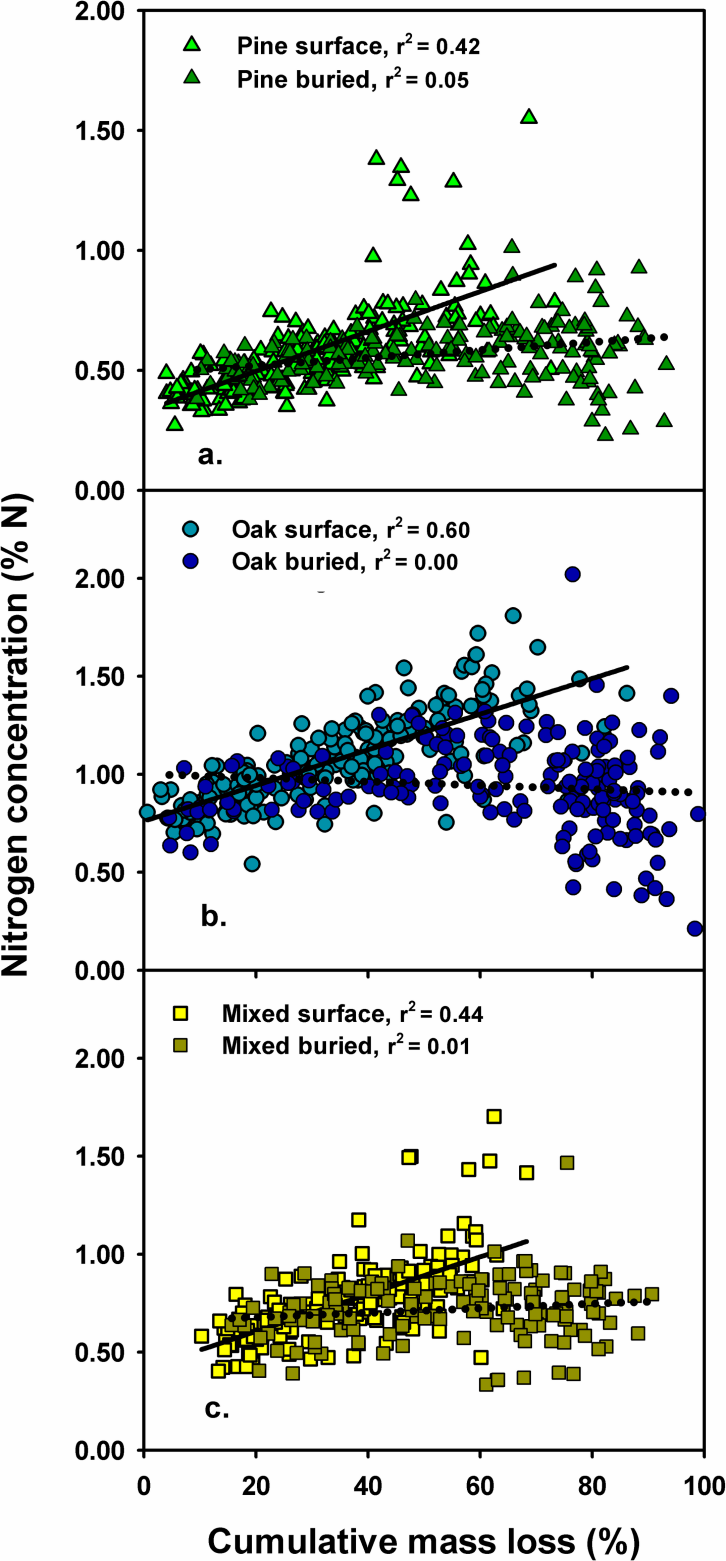
**The relationship between cumulative mass loss and nitrogen concentration in litter in litterbags on the surface of the forest floor and buried beneath pocket gopher mounds over a four year period;** a) longleaf pine litter, b) turkey oak litter, c) mixed pine and oak litter. Complete statistics for the regression lines for surface (solid lines) and buried (dotted lines) litterbags are in S3 Table.

Initial C content in longleaf pine and turkey oak litter was similar, while initial N and P content were 2.0 and 1.4 times greater in oak litter than in pine litter, respectively (Table 1). Mass loss was greater, and N and P were released more rapidly from pine, oak and mixed litter buried beneath pocket gopher mounds than from litter on the surface of the forest floor (Fig 3, Table 2, S2 Table). Burial of litter reduced the estimated time to complete Phase 1 of decomposition (20% mass remaining) by more than half for all litter types; estimated values for buried and surface litter were 3.1 and 8.3 years for pine, 2.1 and 6.7 years for oak, and 2.3 and 6.0 years for mixed litter, respectively. Mass loss was similar from pine, oak and mixed litter on the surface of the forest floor, and initially more rapid from oak and mixed litter than from pine litter beneath pocket gopher mounds (Fig 3A, Table 2, S2 Table). Pine and oak litter on the surface initially immobilized N but mixed litter did not (Fig 3B, Table 2). Oak and mixed litter then released N more rapidly than pine litter in both locations (Fig 3B, Table 2). Accumulated mass loss and N concentration were positively related in litter for pine, oak and mixed litter on the surface of the forest floor, but not for buried oak or mixed litter (Fig 4, S3 Table). Phosphorous also was released more rapidly from buried pine, oak and mixed litter compared to litter on the surface of the forest floor (Fig 3C, Table 2). The amount of P remaining in mixed litter was intermediate between amounts in pine and oak litter in both locations during most sampling periods (Fig 3C, Table 2).

**Table 1:** Initial carbon, nitrogen and phosphorus content of longleaf pine and turkey oak litter in pine, oak and mixed litterbags. Values are means ±1 SE; n = 15 for C and N contents, n = 10 for P content.

Variable	Pine	Oak	Mixed
Carbon (mg C g^-1^)	480.7 ± 2.6	474.2 ± 0.7	477.2 ± 1.8
Nitrogen (mg N g^-1^)	3.6 ± 0.1	7.2 ± 0.2	5.4 ± 0.1
C/N ratio	136.0 ± 3.7	66.4 ± 1.5	88.8 ± 1.2
Phosphorus (mg P g^-1^)	0.22 ± 0.02	0.30 ± 0.03	0.26 ± 0.03

**Table 2:** Results of analyses with linear mixed models for the decomposition parameter (k), nitrogen mass remaining at 24 and 48 months, and phosphorus mass remaining at 24 and 48 months in pine, oak, and mixed litter on the forest floor and buried beneath pocket gopher mounds. Satterthwaite approximation was used to calculate degrees of freedom, df.

Variables	Df	F	*P*
Decomposition coefficient, k			
Litter type	2, 168	66.4	< 0.001
Location^1^	1, 168	1700.9	< 0.001
Litter type * Location	2, 168	5.3	< 0.01
Nitrogen mass remaining after 24 months			
Litter type	2, 121	15.8	< 0.001
Location	1, 61	512.1	< 0.001
Litter type * Location	2, 121	5.8	< 0.01
Nitrogen mass remaining after 48 months			
Litter type	2, 79	22.1	< 0.001
Location	1, 55	113.8	< 0.001
Litter type * Location	2, 79	0.8	N.S.
Phosphorus mass remaining after 24 months			
Litter type	2, 138	0.2	N.S.
Location	1, 135	23.0	< 0.001
Litter type * Location	2, 138	2.5	N.S.
Phosphorus mass remaining after 48 months^2^			
Litter type	1, 86	6.6	< 0.05
Location	1, 86	28.0	< 0.001
Litter type * Location	1, 86	0.1	N.S.

^1^ Location refers to placement of litterbags on the surface of forest floor or beneath pocket gopher mounds.

^2^ This analysis did not include buried mixed litter because of small sample sizes.

**Fig 5:**
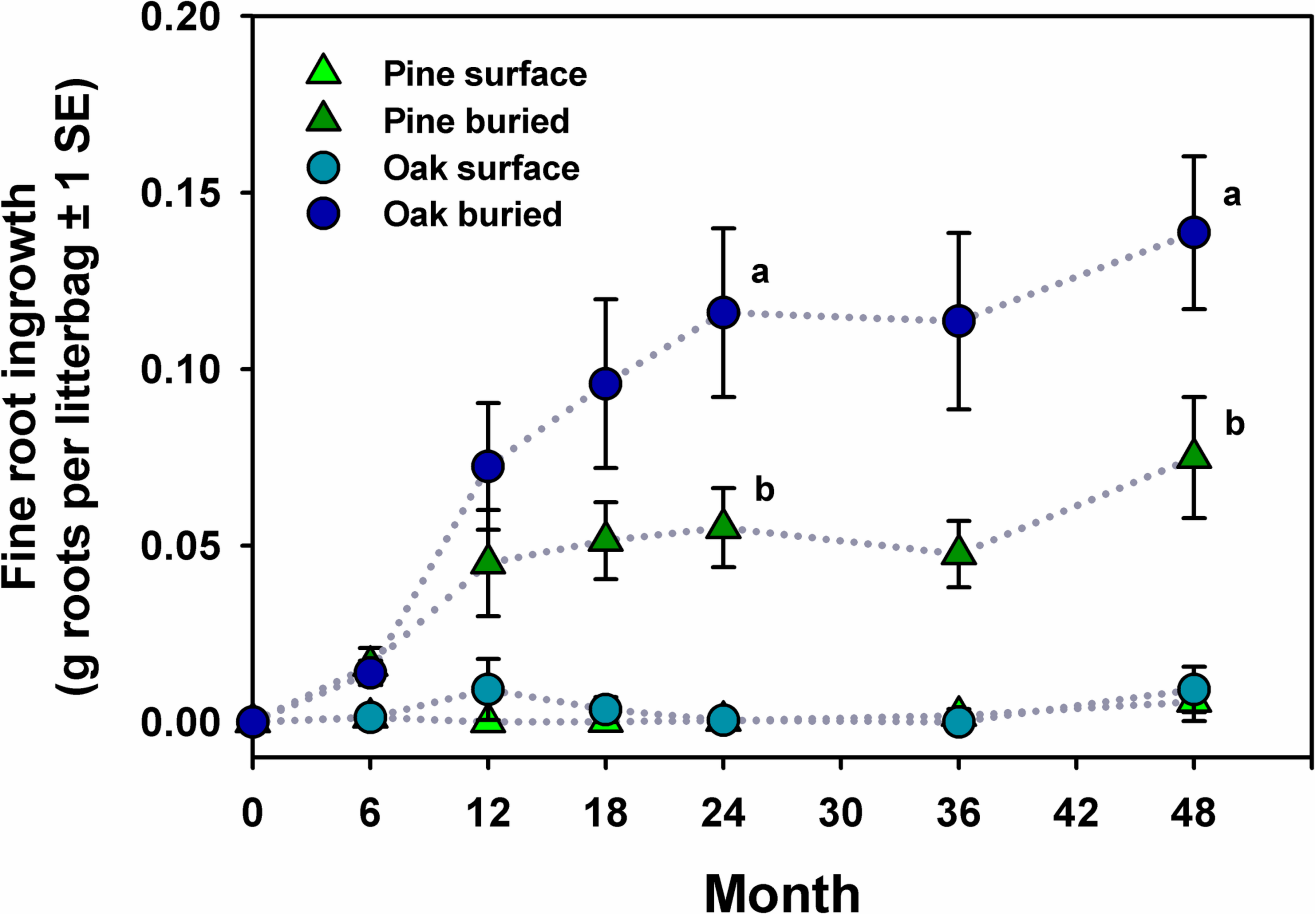
Fine root ingrowth into longleaf pine and turkey oak litter in litterbags on the surface of the forest floor and buried beneath pocket gopher mounds over a four year period. Values are mean g fine roots / litterbag ± 1 SE.

After litterbags had been in the field for 12 months, fine root ingrowth had occurred in most buried litterbags (pine, 75%; oak, 86%), but only in one litterbag on the forest floor (oak) (Fig 5). After 48 months, fine root ingrowth was still much more frequent in buried litterbags (pine, 80%; oak, 95%) than in litterbags on the forest floor (pine, 6%; oak, 18%). Litter type influenced ingrowth of roots into buried litterbags (Fig 5; F_1,316_ = 16.3, *p* < 0.001); ingrowth was significantly greater in oak litter than pine litter at 24 months (*p* < 0.02) and 48 months (*p* < 0.05).

### Simulation of litter layer dynamics

#### No disturbance simulations

Simulated accumulation of pine and oak litter on the surface of the forest floor in the absence of disturbance reached stable values of approximately 780 g m^-2^, 5.8 g N m^-2^, and 0.14 g P m^-2^ after nine years during Phase 1 of decomposition. When we allowed decomposition to proceed past Phase 1 in model simulations, estimated total forest floor and accumulated N and P values stabilized at 925 g m^-2^, 8.6 g N m^-2^, and 0.19 g P m^-1^ after 15 years.

#### Single mound simulation

**Fig 6:**
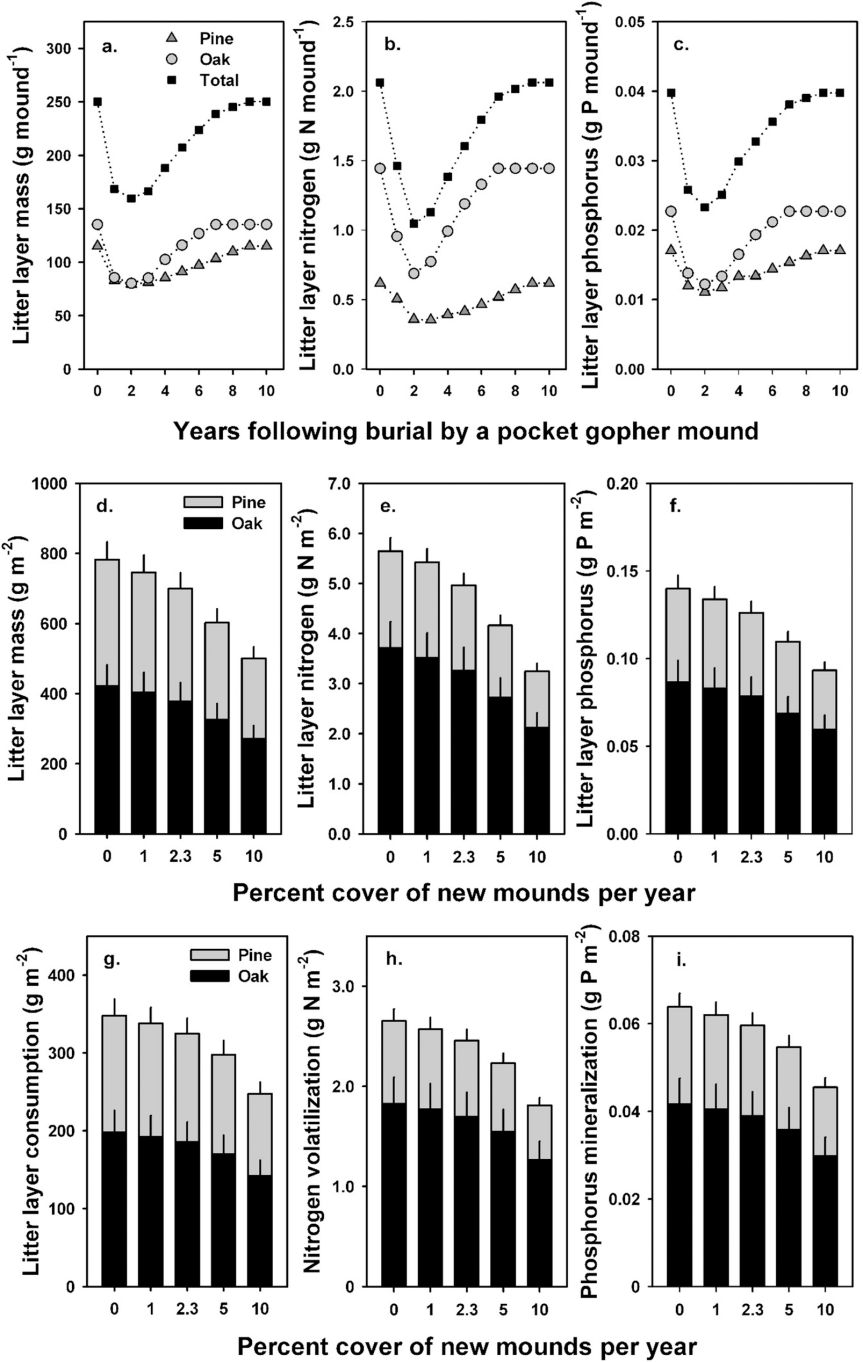
Model simulations of litter mass, nitrogen, and phosphorus content on the forest floor of a longleaf pine forest. (a-c) Litter layer dynamics of an individual pocket gopher mound over a 10–year period predicted by the single mound simulation; (a) mass of pine needle and oak leaf litter, (b) nitrogen in pine needle and oak leaf litter, and (c) phosphorus in pine needle and oak leaf litter. Burial occurred at year zero, and the appropriate litter decomposition rates and average litterfall values were used in simulations. (d-f) Simulated litter layer dynamics of the forest floor as a function of new mound formation at 0%, 1%, 2.3%, 5% and 10% of the forest floor covered per year over an eight–year period predicted by the mound density simulations; (d) mass of pine needle and oak leaf litter, (e) nitrogen in pine needle and oak leaf litter, and (f) phosphorus in pine needle and oak leaf litter. Vertical lines above each bar indicate variation (± 1 SE) in the amount of litterfall mass, nitrogen and phosphorus. (g-i) Simulated litter layer consumption, nitrogen volatilization, and phosphorus pyro-mineralization during low-intensity fires occurring at a five-year return interval predicted by the mound density and fire simulations; (g) consumption of pine needle and oak leaf litter, (h) nitrogen volatilization from pine needle and oak leaf litter, and (i) phosphorus pyro-mineralization from pine needle and oak leaf litter. Simulated rates of new mound formation were 0%, 1%, 2.3%, 5% and 10% of the forest floor covered per year. Vertical lines above each bar indicate variation (± 1 SE) in the amount of litterfall mass, nitrogen or phosphorus.

Simulation of an individual pocket gopher mound indicates that minimum values of foliar litter mass and N and P content of the litter layer occurred approximately two years following burial even though litter continued to accumulate on top of the mounds. Minimum values for mass, N, and P content were 36%, 49%, and 41% lower than values for the litter layer in areas with no mounds predicted in the no disturbance scenario, respectively (Fig 6A–6C). Minimum values were largely driven by the relatively rapid release of C, N and P from turkey oak litter. Litter layer mass and N and P content then increased as litter continued to accumulate on top of the mound through time, and pre-burial values equivalent to predictions by the no disturbance scenario were achieved within nine years following burial (Fig 6B and 6C).

#### Mound density simulations

Simulated litter layer mass and N and P content decreased with an increase in the rate of new pocket gopher mound formation from no mounds to a 10% cover of new mounds per year (Fig 6D–6F). At a rate of new mound formation that resulted in 2.3 ± 0.6% cover of the forest floor per year, predicted mass and accumulated N and P content in pine and oak litter on the forest floor over a nine–year period were 11%, 12% and 10% less than those predicted by the no disturbance simulation, respectively. At the highest simulated annual rate of new mound formation covering 10% of the forest floor per year, which was more representative of local densities (e.g., Fig 1B), litter layer mass and accumulated N and P averaged 36%, 42% and 33% less than values predicted by the no disturbance simulation, respectively. These latter values for N and P turnover represent an additional cumulative release of 2.4 g N m^-2^ and 0.05 g P m^-2^ over an eight–year period, equivalent to 29% and 17% of N and P flux in foliar litterfall from the canopy over the same period, respectively.

#### Prescribed fire simulations

Over a 25–year simulation and assuming minimal change to litterfall amounts and composition, a simulated fire return interval of five years that consumed approximately 50% of the litter layer resulted in minimum fine litter mass of 430 g m^-2^ immediately following a burn and a maximum of 720 g m^-2^ just prior to the next burn, following a 12–year equilibration period. Minimum and maximum values for N and P content of the litter layer ranged from 2.7 to 5.3 g N m^-2^ and 0.06 to 0.13 g P m^-2^, respectively, assuming that N was volatized in proportion to litter consumption, and that pyro-mineralized P remaining in the ash layer immediately following prescribed fires was assimilated by microbial biomass and plants or leached from the litter layer.

#### Mound density and fire simulations

**Fig 7:**
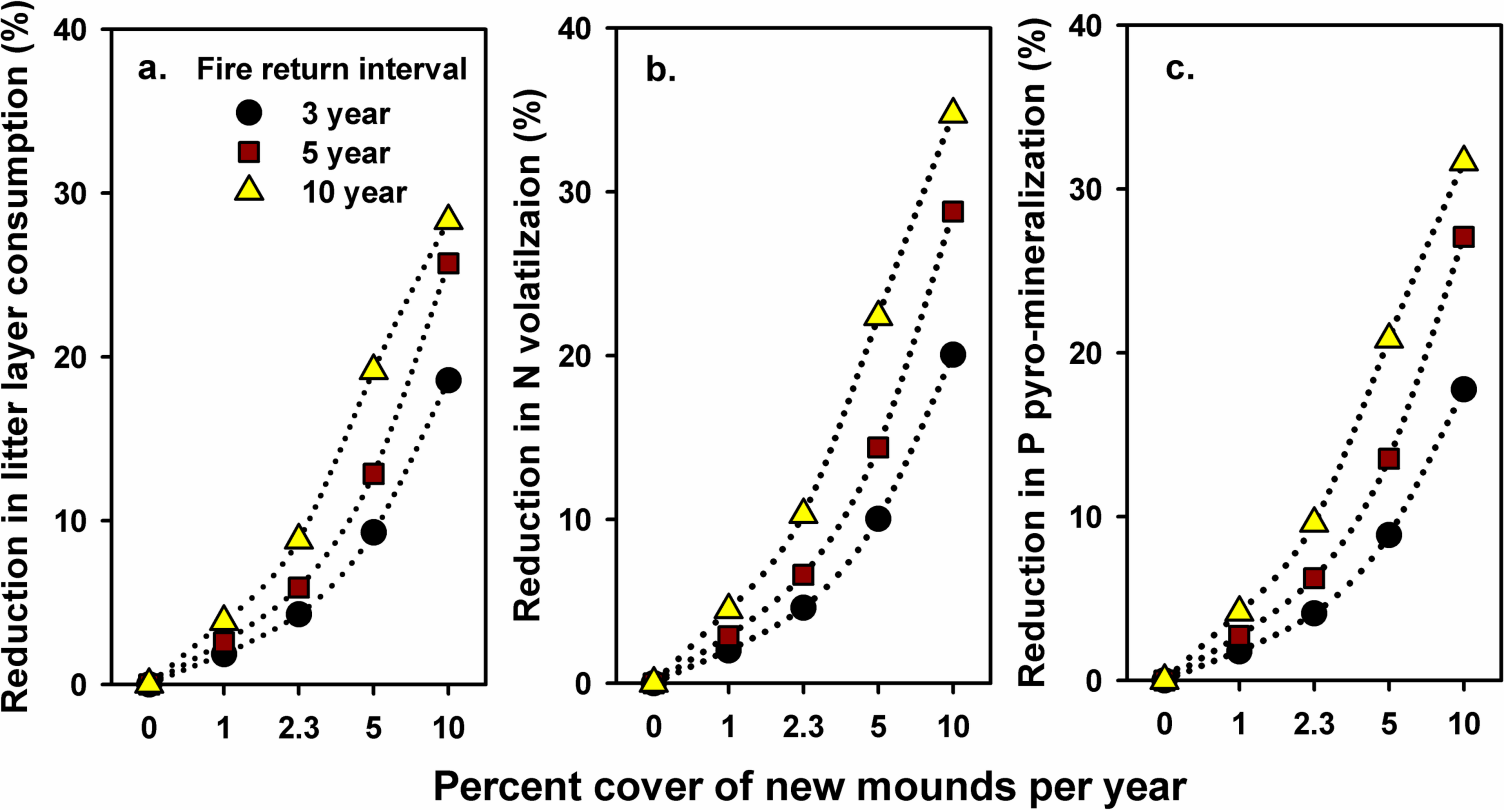
Simulated mass consumption, nitrogen volatilization, and phosphorus pyro-mineralization of the litter layer at three fire return intervals and five rates of pocket gopher mound formation. Simulated fire return intervals are 3, 5 and 10 years, and rates of new mound formation are 0%, 1%, 2.3%, 5% and 10% of the forest floor covered per year. Values are percent reduction of (a) mass consumption, (b) N volatilization, and (c) P pyro-mineralization predicted by the mound density and fire simulations compared to prescribed fire only simulations.

Increasing the rate of pocket gopher mound formation from no mounds to 10% cover of new mounds per year reduced predicted consumption of the litter layer and decreased volatilization of N and pyro-mineralization of P (Fig 6G–6I). Greater amounts of litter are protected from consumption with increasing density of new mounds, and reduced amounts of litter are available for consumption on previously formed mounds. At a rate of new mound formation that covers 2.3% of the forest floor per year and a fire return interval of five years, predicted litter layer consumption, N volatilization and P pyro-mineralization were 7%, 8% and 7% less than values predicted by the prescribed burn simulation in the absence of pocket gopher mounds, respectively (Fig 6G–6I). An increase in the fire return interval from 3 to 10 years resulted in reduced consumption, N volatilization and P pyro-mineralization at all densities of new mound formation because mounds covered greater amounts of accumulated litter on the forest floor as fire interval increased (Fig 7A–7C).

## Discussion

Our study provides evidence for interactions of ecosystem engineering and fire that result in alternating acceleration and deceleration of N and P turnover in the longleaf pine ecosystem. Burial of the forest floor by pocket gophers increases rates of litter decomposition and nutrient turnover, creating patches of enhanced nutrient supply. Vascular plants respond with increased growth of fine roots into buried litter, some of which are colonized by mycorrhizal fungi. When fires occur, buried forest floor material is protected from consumption and, thus, activities of pocket gophers reduce N volatilization and particulate transport, major pathways of nutrient loss from this ecosystem. This interaction of ecosystem engineering and fire promotes conservation of N, P and other nutrients in the longleaf pine ecosystem, where productivity is thought to be limited by N and P availability [31,34,56].

Mass loss from plant litter during the decomposition process is an integration of litter comminution, leaching of soluble compounds and small particles, and gaseous losses of carbon dioxide as a result of microbial respiration [47,52]. Rapid mass loss from buried litter compared to litter on the surface of the forest floor indicates that a more favorable environment for microbial activity occurs beneath pocket gopher mounds. Higher surface temperatures and lower moisture contents occur near the surface of recently formed pocket gopher mounds compared to unburied litter layer [57], but burial is likely to buffer temperature and moisture extremes at the depth of the forest floor. Burial also facilitates root ingrowth and mycorrhizal colonization in buried litter as decomposition progresses. Nitrogen dynamics during decomposition of plant litter has been characterized as a three-phase process; initial leaching of soluble N, net immobilization of N by microbial populations colonizing litter, and then net N release as C in litter continues to be respired [51,58]. This pattern was observed for pine and oak litter on the forest floor and for buried pine litter, but net immobilization of N by buried oak or mixed litter was minimal, and net N release from buried pine and oak litter began relatively early in the decomposition process. The universally observed pattern where N concentration increases linearly with progressive mass loss as decomposition proceeds (e.g., [51,58,59]) was not observed for buried oak or mixed litter. Overall, N and P dynamics in decomposing litter on the surface of the forest floor in our study were similar to other pine-dominated forests in the southeastern US, but N and P were released more rapidly from litter buried beneath pocket gopher mounds compared to unburied litter in other studies [52,56,60] (S4 Table).

During low-intensity prescribed fires in long leaf pine stands, litter consumption is proportional to initial litter mass on the forest floor, with an average of approximately 52 to 75% of fine litter consumed [53–55], similar to other pine dominated stands on the Atlantic Coastal Plain (e.g., [61]). Loss of N by volatilization and particulate transport can represent up to 80 to 90% of the N in litter layer and understory vegetation during prescribed burns [32–34]. Forest floor material buried by pocket gopher mounds is protected from consumption by fire, reducing N volatilization and loss by other processes. As fire intervals increase, new pocket gopher mounds cover more years of accumulated litter, and thus sequester a larger proportion of the litter from combustion. Recently formed pocket gopher mounds also result in patches of bare soil and introduce variation in fuel loading, producing discontinuities in fuel bed that may have significance for fire behavior and subsequent fire effects [30,62–64].

Research on effects of pocket gophers on nutrient cycling largely has been conducted in herb-dominated ecosystems (e.g., [10,14,15,65]). Acceleration of nutrient turnover in these systems occurs through a similar process as in our forested system (i.e., burial of litter), but deceleration mechanisms differ. Following an initial increase in N mineralization in herb-dominated ecosystems, N turnover rates often decrease because burrowing activities and foraging by pocket gophers reduce herbaceous plant biomass and subsequent litter production [10,15,65]. Bioturbation in woodlands and forests does not directly interfere with productivity of mature woody vegetation, the source of most litter, and, thus, litter production and N and P inputs to the forest floor are largely unaffected by these animal activities. In contrast, the interaction of fire and litter burial by pocket gophers, and potentially other bioturbators, is an important mechanism of deceleration of nutrient turnover and loss in longleaf pine ecosystems, and likely in other woodland and forested systems where fire and animal activities that cover the forest floor with soil are both common [7].

Longleaf pine forests have one of the highest levels of forb and grass diversity of forested ecosystems in North America [21–23]. Accumulated litter and humus is a primary factor regulating understory plant diversity because of interference with seed germination, seedling establishment and regeneration by sprouting, thus processes that create heterogeneity in the litter layer contribute to the maintenance of diversity in this system [21,36,66]. This has led to a focus on fire behavior in management and restoration efforts, and recognition of the importance of fine-scale heterogeneity in the distribution of fuels on the forest floor [30,62–64,67]. Herbivory is the primary process by which animals are known to alter fuel loads and fire behavior [4,68,69]. However, our study demonstrates that pocket gophers reduce fuel loads and introduce spatial and temporal heterogeneity on the forest floor through other mechanisms, and that this heterogeneity is magnified in the presence of fire. As with fire, soil excavation and ejection by pocket gophers produce patches of bare mineral soil, which are important in seedling germination and establishment [10,70–72]. However, in contrast to the low nutrient of patches of mineral soil created when fire consumes organic matter and N is lost by volatilization, bare patches produced by bioturbation contain underlying organic matter that has high nutrient availability and greater water holding capacity. At very high return frequencies, fire can reduce spatial variability in fuel loads by repeatedly consuming fuels and, thus, have a homogenizing effect on fine scale heterogeneity [27,53,61]. However, even when frequent fires occur, mounding activities maintain fine scale heterogeneity by conserving patches with relatively high resource availability under mounds. Finally, when mounds are dense, pocket gophers have the potential for landscape scale effects on fire behavior by producing fuel discontinuities that function as fire breaks, thus creating heterogeneity at a larger scale, as has been demonstrated for bioturbators in fire-prone systems of Australia [7].

Restoration efforts in longleaf pine ecosystems, as in other systems, rely on considerable human intervention to restore ecosystem structure and species diversity (e.g., repeated prescribed fires and selective silvicultural treatments) [21,36,37,73,74]. By altering interactions between biotic and abiotic processes, pocket gophers and other ecosystem engineers can produce one of the target outcomes of restoration for longleaf pine ecosystems, increased heterogeneity of the litter layer, while simultaneously conserving limiting nutrients that potentially are depleted during frequent fires. Ecosystem engineers that disturb soil and increase heterogeneity in fuel loads have been shown to be important in ecosystem structure, productivity and fire effects in a wide range of fire-prone systems [4,7,11,16,68]. Because populations of many of these ecosystem engineers are locally extinct or in decline, their important roles in ecosystem functioning are diminished. Thus, targeted conservation and reintroduction of these species might be integral to successful restoration efforts [7,39,43,75].

## Supporting information

S1 Table Annual litterfall mass (g m^-2^ year^-1^) collected over a three year period at the Ordway-Swisher biological station. (DOCX)

S2 Table Decay constants (k, mean ± 1 SE) and statistics for model fit for mass loss from litter of longleaf pine, turkey oak, and mixed pine and oak litter on the surface of the forest floor and buried beneath pocket gopher mounds. Data were fit to an exponential decay model (remaining mass = e−^k * time^). k values with different letters are significantly different (p < 0.05). Estimated average time to the end of Phase 1 of decomposition, defined as < 20% original litter mass remaining, is also presented.
(DOCX)

S3 Table Model parameters and statistics for the relationship between cumulative mass loss (%) and N concentration in pine, oak and mixed litter on the surface of the forest floor and buried under pocket gopher mounds. All data were fit to N concentration = α (percent cumulative mass loss) + β.
(DOCX)

S4 Table Initial litter composition and decomposition statistics for litterbag studies in pine-dominated stands in the Southeastern U.S. Decomposition coefficients (k) are calculated on an annual basis.
(DOCX)

S1 Appendix R program used for statistical analyses. (DOCX)

S2 Appendix Description of the simulation models. (DOCX)
