# Supplementary material for: Bioturbation by mammals and fire interact to alter ecosystem-level nutrient dynamics in longleaf pine forests
Source: PLoS One. 2018 Aug 22;13(8):e0201137. doi: 10.1371/journal.pone.0201137 (PMC6104935; doi:10.1371/journal.pone.0201137)
Supplement: S1 Table — (DOCX) [file pone.0201137.s001.docx]

| Litter type | Year 1 | Year 2 | Year 3 |
| --- | --- | --- | --- |
| Pine needles | 71.8 ± 10.4 | 76.0 ± 11.4 | 86.2 ± 11.2 |
| Oak foliage | 117.8 ± 16.9 | 114.6 ± 15.8 | 99.3 ± 14.5 |
| Other foliage | 0.4 ± 0.6 | 0.7 ± 1.8 | 1.5 ± 2.4 |
| Wood | 32.7 ± 11.6 | 17.0 ± 6.9 | 28.3 ± 10.7 |
| Miscellaneous | 26.0 ± 4.2 | 29.5 ± 6.3 | 33.3 ± 6.0 |

**S1 Table. Annual litterfall mass (g m^-2^ year^-1^) collected over a three year period at the Ordway-Swisher Biological Station.**
