# Supplementary material for: Bioturbation by mammals and fire interact to alter ecosystem-level nutrient dynamics in longleaf pine forests
Source: PLoS One. 2018 Aug 22;13(8):e0201137. doi: 10.1371/journal.pone.0201137 (PMC6104935; doi:10.1371/journal.pone.0201137)
Supplement: S2 Table — Data were fit to an exponential decay model (remaining mass = e−k * time). k values with different letters are significantly different (p < 0.05). Estimated average time to the end of Phase 1 of decomposition, defined as < 20% original litter mass remaining, is also presented. (DOCX) [file pone.0201137.s002.docx]

| Litter type | Location | k | F | r^2^ | *P* | Phase 1 |
| --- | --- | --- | --- | --- | --- | --- |
| Longleaf pine | Surface | 0.194 ± 0.004^a^ | 850.7 | 0.993 | <0.001 | 8.3 |
|  | Buried | 0.529 ± 0.021^b^ | 146.1 | 0.960 | <0.001 | 3.1 |
| Turkey oak | Surface | 0.241 ± 0.006^a^ | 558.2 | 0.989 | <0.001 | 6.7 |
|  | Buried | 0.775 ± 0.039^c^ | 90.7 | 0.937 | <0.001 | 2.1 |
| Mixed | Surface | 0.268 ± 0.006^a^ | 72.4 | 0.923 | <0.001 | 6.0 |
|  | Buried | 0.704 ± 0.017^c^ | 50.9 | 0.893 | <0.001 | 2.3 |

**S2 Table. Decay constants (k, mean ± 1 SE) and statistics for model fit for mass loss from litter of longleaf pine, turkey oak, and mixed pine and oak litter on the surface of the forest floor and buried beneath pocket gopher mounds.** Data were fit to an exponential decay model (remaining mass = e ^–k * time^). k values with different letters are significantly different (p < 0.05). Estimated average time to the end of Phase 1 of decomposition, defined as < 20% original litter mass remaining, is also presented.
