# Supplementary material for: Bioturbation by mammals and fire interact to alter ecosystem-level nutrient dynamics in longleaf pine forests
Source: PLoS One. 2018 Aug 22;13(8):e0201137. doi: 10.1371/journal.pone.0201137 (PMC6104935; doi:10.1371/journal.pone.0201137)
Supplement: S3 Table — All data were fit to N concentration = α (percent cumulative mass loss) + β. (DOCX) [file pone.0201137.s003.docx]

**S3 Table. Model parameters and statistics for the relationship between cumulative mass loss (%) and N concentration in pine, oak and mixed litter on the surface of the forest floor and buried under pocket gopher mounds.** All data were fit to N concentration = α (percent cumulative mass loss) + β.

| Litter type | Location | α | β | r^2^ | F | *p* |
| --- | --- | --- | --- | --- | --- | --- |
| Longleaf pine | Surface | 0.008 ± 0.001 | 0.330 ± 0.023 | 0.42 | 147.1 | <0.001 |
|  | Buried | 0.002 ± 0.001 | 0.487 ± 0.029 | 0.05 | 10.6 | <0.05 |
| Turkey oak | Surface | 0.009 ± 0.001 | 0.761 ± 0.020 | 0.60 | 313.9 | <0.001 |
|  | Buried | -0.001 ± 0.001 | 1.001 ± 0.050 | 0.00 | 1.7 | N.S. |
| Mixed | Surface | 0.010 ± 0.001 | 0.415 ± 0.031 | 0.44 | 151.1 | <0.001 |
|  | Buried | 0.001 ± 0.001 | 0.655 ± 0.038 | 0.01 | 3.2 | N.S. |
