# Supplementary material for: Bioturbation by mammals and fire interact to alter ecosystem-level nutrient dynamics in longleaf pine forests
Source: PLoS One. 2018 Aug 22;13(8):e0201137. doi: 10.1371/journal.pone.0201137 (PMC6104935; doi:10.1371/journal.pone.0201137)
Supplement: S4 Table — Decomposition coefficients (k) are calculated on an annual basis. (DOCX) [file pone.0201137.s004.docx]

**S4 Table. Initial litter composition and decomposition statistics for litterbag studies in pine-dominated stands in the Southeastern U.S.**  Decomposition coefficients (k) are calculated on an annual basis.

| Site/Litter type | Initial composition | | | Decomposition statistics | |
| --- | --- | --- | --- | --- | --- |
|  | Carbon | Nitrogen | Phosphorus | k | r^2^ |
|  | % | mg g^-1^ | |  |  |
| This study, surface litter^1^ | | | | | |
| Longleaf pine | 48.1 | 3.6 | 0.22 | 0.194 | 0.99 |
| Turkey oak | 47.4 | 7.2 | 0.30 | 0.241 | 0.99 |
| Mixed | 47.7 | 5.4 | 0.26 | 0.268 | 0.92 |
| Georgia, USA^2^ |  |  |  |  |  |
| Longleaf pine | 52.0 | 3.5 | 0.17 | 0.113 | 0.94 |
| Chestnut oak | 51.5 | 6.9 | 0.21 | 0.136 | 0.94 |
| N. Florida, USA^3,4^ |  |  |  |  |  |
| Slash pine | 54.3 | 3.6 | 0.19 | 0.138 | 0.94 |
| Red pine | 52.8 | 5.9 | --- | 0.227 | 0.90 |

^1.^1-mm mesh size litterbags; ^2^ [56], 2-mm mesh size litterbags; ^3^[60], average of 27 and 35 year old stands, litterbags with 1 mm mesh size on the top and 0.055 mm mesh on the bottom; ^4^[52], litterbags with 1-mm mesh size on the top and 0.055 mm mesh on the bottom, litter carbon content from LIDET study.
