# Supplementary material for: Bioturbation by mammals and fire interact to alter ecosystem-level nutrient dynamics in longleaf pine forests
Source: PLoS One. 2018 Aug 22;13(8):e0201137. doi: 10.1371/journal.pone.0201137 (PMC6104935; doi:10.1371/journal.pone.0201137)
Supplement: S1 Appendix — (DOCX) [file pone.0201137.s005.docx]

**S1 Appendix: R Code used for analyses of the litterbag**

**decomposition study.**

# R Code for analysis for pocket gopher litter bag decomposition study

# R Code below is for P at 24 months with log transformed data.

# Same code was used for: 1) N at 24 and 48 months, data not transformed;

# 2) K values, log-transformed; and 3) root in-growth in buried bags, data

# not transformed, but location (buried vs.forest floor) not included

library(car)

library(lme4)

library(lmerTest)

library (lsmeans)

library(plotrix)

library(dplyr)

setwd("~/1_ufbiz/research/FL pocket gophers/Analysis N_P 2018")

# Data are phosphorus values log transformed for each plot in each block for

# buried and unburied bags. Bags collected at 24 months.

data=read.csv("Final P 24 mo.csv", header = TRUE)

# Checks on data structure

# Lists variables in data

names(data)

# Lists structure of the data

str(data)

# Lists names of levels for each factor

levels(data$Litter)

levels(data$Location)

# Location is buried or forest floor

levels(data$Block)

# Assign variable to be a factor not a numeric variable

data$Sample_Plot = as.factor(data$Sample_Plot)

data$Months = as.factor(data$Months)

# Transform data (for k value and P content at 24 months)

data$Log_Perc_P = log(data$Perc_P)

# Model run with lme4 package, includes location nested within sample_plots

# and sample_plots nested within blocks

lme_P_model <- lmer(Log_Perc_P ~ Litter + Location + Litter*Location + (1|Block/Sample_Plot/Location),data)

summary (lme_P_model)

anova(lme_P_model, type = 3)

lsm_P<-lsmeansLT(lme_P_model, test.effs=NULL)

lsm_P

difference_lsm_P<-difflsmeans(lme_P_model,test.effs=NULL)

difference_lsm_P

lsm_P2<-lsmeans(lme_P_model, list (pairwise ~ Litter*Location))

cld(lsm_P2)
