# Supplementary material for: Bioturbation by mammals and fire interact to alter ecosystem-level nutrient dynamics in longleaf pine forests
Source: PLoS One. 2018 Aug 22;13(8):e0201137. doi: 10.1371/journal.pone.0201137 (PMC6104935; doi:10.1371/journal.pone.0201137)
Supplement: S2 Appendix — (DOCX) [file pone.0201137.s006.docx]

**S2 Appendix: Simulation model description**

In this supplement we describe the models used to simulate litter layer mass and nitrogen (N) and phosphorus (P) dynamics of the litter layer in a longleaf pine forest at the Ordway Swisher Biological Station. The organization of this supplement is; 1) a brief overview of the models, 2) a description of model inputs and related variables necessary to simulate each scenario, 3) model equations and calculations, 4) a description of model outputs, 5) a detailed explanation of simulation scenarios and their assumptions, and 6) additional references.

**Modeling Overview**

We developed a set of spreadsheet simulation models in Excel in the Microsoft Office 2013 suite (Microsoft Corp., Redmond, WA, USA) to predict the dynamics of pine needle and oak leaf litter mass and nitrogen (N) and phosphorus (P) content in the litter layer of a longleaf pine forest. The simulations are used to explore the effects of litter burial by pocket gopher mounds and the interactive effects of litter burial and low intensity prescribed fires on the net accumulation of mass, N, and P. Average annual fluxes of pine needle and oak leaf litter that were collected in the field over a three–year period and initial average weighted mean concentrations of N and P in litter are used as the primary model inputs. Net changes to annual “cohorts” of litter during the decomposition process are predicted based on an exponential decay function using parameters for surface or buried litter derived from the 48–month litterbag decomposition study. Nitrogen and P content in decomposing litter is calculated using the relationships between remaining litter mass and N and P concentrations, also derived from the litterbag study. The models predict the mass and N and P content of pine and oak litter on an annual time step by updating values for annual litter “cohorts” every year. Nitrogen and P dynamics of surface and buried litter were simulated separately because the litterbag study indicated that fundamental patterns of nutrient release during decomposition process were altered by burial under pocket gopher mounds, with no net immobilization of N occurring in buried oak litter, and very little net N immobilization in buried pine litter compared to surface litter. Further, pine and oak litter are simulated separately because their initial N and P concentrations and dynamics differed on the surface of the forest floor and buried beneath pocket gopher mounds. Values for annual cohorts of each type of litter are then summed to estimate mass and N and P content of the litter layer every year. Five scenarios were simulated: 1) litter layer dynamics in the absence of pocket gopher mounds or prescribed fire (no disturbance simulations), 2) litter layer dynamics of an individual mound (single mound simulation), 3) litter burial by pocket gophers at five annual rates of new mound formation (mound density simulations), 4) low-intensity prescribed fires conducted at periodic intervals (prescribed fire simulations), and 5) interactive effects of litter burial and low-intensity fire (mound density and fire simulations).

**Model Inputs**

Model inputs are 1) the annual average litterfall of longleaf pine needles and turkey oak leaves in the three areas shown in Fig 2 and S1 Table, in units of g m^-2^ year^-1^, and 2) average weighted mean N and P concentrations in litter derived from litterfall measurements in the three areas shown in Table 1, in units of g N g litter^-1^ or g P g litter^-1^. Three other variables necessary for specific model simulation scenarios are; 1) the average area of forest floor covered by an individual mound (0.32 m^2^) for the single mound simulation, 2) the percent of forest floor covered by newly formed mounds per year for the mound density simulations and the mound density and fire simulations, and 3) the annual return interval of prescribed fires in the prescribed fire simulations and the mound density and fire simulations. Lastly, a key relationship used to estimate N and P concentrations in decomposing litter in all simulations is based on the relationships between mass loss and N and P concentrations in oak or pine litter on the surface of the litter layer or buried beneath pocket gopher mounds derived from the litterbag study. These relationships for N are shown in Fig 4 and parameters are presented in S3 Table.

**Model calculations and equations**

Central to our simulations are three equations, the first used to calculate litter mass remaining at a specified time, the second to calculate N and P content in remaining litter each year, and the third to calculate litter layer consumption during low intensity prescribed fires. The first equation is the exponential decay model derived by Olson [47], with the form:

$Mass remaining = e^{-k*time}$ (1)

Where mass remaining is a fraction of initial mass, k is the negative exponential decay constant, and time is time in years. Separate exponential decay constants derived from litter decomposition data for pine needles or oak leaves in litterbags on the surface of the forest floor or buried beneath pocket gopher mounds were used to simulate mass loss from litter in the appropriate location. Values for k used for all simulations are shown in S2 Table. Needle and leaf mass remaining on the forest floor or buried beneath pocket gopher mounds were predicted for each annual “cohort” of litter by multiplying the mass remaining term by average litterfall mass for surface litter (g mound^-1^ or g m^-2^). We predicted litter layer mass at the beginning of each calendar year after the majority of oak and pine litter had fallen, and assumed that no net loss of N or P had occurred from the current year litter cohort at time = 0.

To simulate N and P dynamics during the decomposition process, a second equation that characterizes the relationships between percent litter mass remaining and N and P concentrations derived from the litterbag study was used to calculate the content of N or P in each annual cohort of decomposing litter:

$N concentration \left( t \right)= N concentration \left( t = 0 \right)*\alpha\left( \% mass remaining \left( t \right) \right)+ \beta$ (2)

Where t is time in years, and α and β are the slope and intercept estimated from the relationship between percent mass remaining and N or P concentrations for pine needles or oak leaves on the surface of the litter layer, or buried beneath pocket gopher mounds. Values for α and β were derived empirically from the litterbag study. This relationship for N is shown in Fig 4 and S3 Table, and similar relationships for pine needle and oak leaf litter were calculated for P.

For the prescribed fire simulations, we used a third equation to calculate consumption of annual cohorts of litter. We assumed that 50 % of the litter layer was consumed, consistent with pre- and post-burn measurements in longleaf pine forests [53–55]. Consumption was calculated as a proportion of each annual litter cohort:

$Litter layer mass (t) = litter layer mass (t-1) * 0.5$ (3)

Where t is time in years. We assumed that 50 % of all annual litter cohorts during Phase 1 of decomposition with > 20% mass remaining were consumed during low intensity prescribed fires, and annual cohorts with < 20% mass remaining were not consumed. We realize that this is only an approximation of litter consumption on the forest floor during prescribed burns, because it is likely that greater amounts of more recent annual cohorts of litter are differentially consumed on the forest floor during prescribed fires. However, because N content (as opposed to N concentration) is similar in successive annual litter cohorts as decomposition progresses on the surface of the litter layer during Phase 1, this approximation results in only minor differences in predicted amounts of N volatilized.

**Model outputs**

Model outputs were annual net accumulation of mass, N and P by pine needle and oak leaf litter in each annual cohort. Annual litter cohorts were summed to estimate mass and N and P remaining in the litter layer on the surface of the forest floor or beneath and on the top of pocket gopher mounds through time. When we simulated only Phase 1 of decomposition products, annual litter “cohorts” that were less than 20% of initial mass were not included in annual summed values for the litter layer. Similarly, N and P in litter cohorts that were less than 20% of initial litter mass were not included in annual sums. Further processing of model outputs included calculations of a percent change from simulations without mounds. For example, results from the mound density and fire simulations were presented as a percent reduction in litter layer consumption as a function of the formation rate of new mounds on the forest floor.

**Model simulation scenarios**

We simulated five scenarios; 1) litter layer dynamics in the absence of pocket gophers or prescribed fire (no disturbance simulations), 2) litter layer dynamics of an individual mound over a 10–year time period (single mound simulation), 3) litter burial by pocket gophers at five annual rates of new mound formation (mound density simulations), 4) low-intensity prescribed fires conducted at periodic intervals using previously published values for forest floor consumption in longleaf pine forests (prescribed fire simulations), and 5) interactive effects of litter burial and low-intensity fire on mass, N and P dynamics of the litter layer (mound density and fire simulations).

**No disturbance simulations**

No disturbance simulations were used to predict litter layer mass and N and P dynamics in the absence of pocket gopher mounds or prescribed burns. We predicted litter layer mass at the beginning of each calendar year, and assumed that current-year litter had fallen completely, consistent with seasonal litterfall measurements. We also assumed that no net loss of N or P from the current year litter cohort had occurred at time = 0. We initially simulated a 25–year period to estimate when Phase 1 decomposition products, defined as 20% of initial mass of an annual litter cohort remaining [51,52], stabilized on the forest floor. We then extended simulation results beyond Phase 1 to estimate when all organic matter derived from the net accumulation of pine needle and oak leaf litter stabilized. Model predictions for accumulated Phase 1 decomposition products averaged 780 g m^-2^ at “steady state” conditions after nine years, and were within the range of forest floor mass measured in other intermediate age longleaf pine stands (265 to 1350 g m^-2^ [76–78]). Phase 1 N and P content of the litter layer totaled 5.8 g N m^-2^ and 0.14 g P m^-2^. When we allowed decomposition to proceed past Phase 1 during simulations, estimated total forest floor and accumulated N and P values stabilized at 925 g m^-2^, 8.6 g N m^-2^, and 0.19 g P m^-2^ after 15 years. Simulated forest floor mass was similar to the average maximum forest floor mass value of 896 ± 75 g m^-2^ estimated for a chronosequence of 30 longleaf pine stands in the southeastern US [78], and much lower than values reported for mixed pine flatwood stands in the southeastern US (2904 ± 216 to 5192 ± 519 g m^-2^ [78–81].

**Single mound simulation**

We simulated litter decomposition and N and P dynamics of pine needle and oak leaf litter at an individual pocket gopher mound to evaluate how burial of the litter layer and subsequent accumulation of litter on top of the mound altered forest floor dynamics, and to determine when Phase 1 of decomposition at mounds returned to “steady state” conditions comparable to values predicted by the no disturbance simulation. For initial conditions, we assumed that the amount and N and P content of litter buried by the mound reflected ‘steady state” for Phase 1 decomposition predicted by the no disturbance simulation, and that the mound was average in size and covered 0.32 m^-2^ on the forest floor. Mound formation was assumed to have occurred after all of the pine needle and oak leaf litter fell in late fall and early winter, consistent with the seasonal peak in mound formation that we observed in the field. A similar seasonal peak in pocket gopher mound formation was reported in [26]. We assumed that the current year cohort of litter reflected mean annual litter fall amounts and N and P content, and decomposed as buried litter did during the litter bag experiment. We also assumed that burial accelerated the decomposition of litter cohorts from previous years, and used k values for buried pine and oak litter for previous year cohorts, corrected for the proportional amount of litter remaining in each annual cohort. For example, pine and oak litter cohorts from previous years were estimated to average 82 and 79% mass remaining for year one, 68 and 62 % for year two, and 56 and 49% for year three, respectively. For N and P contents of litter cohorts from previous years at burial, we used the relationship shown in Figure 4 and S3 Table to estimate N concentrations, and a similar function for P concentrations. We then multiplied values for mass remaining by the appropriate concentrations of N and P. To simulate litter that accumulated on top of the mound in the years following burial, we used average annual litterfall amounts and N and P fluxes that would fall in an area equivalent to the average mound size measured in the field. Decomposition and N and P dynamics were assumed to be equivalent to those measured in surface litter bags, and followed predictions of the no disturbance simulation above. We then simulated the decomposition process to Phase 1, thus when an annual litter cohort had less than 20% of initial mass remaining it was excluded from integrated values. Values from the four types of annual litter cohorts (buried pine, buried oak, surface pine and surface oak) were then summed for each year simulated.

**Mound density simulations**

We used mound density simulations to evaluate how the density of recently formed pocket gopher mounds alters litter layer mass and the dynamics of N and P in litter. Because pocket gophers have a clumped distribution, rates of mound formation and cover of the forest floor by mounds vary widely across the landscape (e.g., Fig 1). We simulated five annual rates of new mound formation, covering no (0% yr^-1^), low (1% yr^-1^), medium-low (2.3% yr^-1^), medium-high (5% yr^-1^), and high (10% yr^-1^) area of the forest floor per year. We assumed that all mounds were the average size measured in the field (0.32 m^-2^) and that no overlap of mounds occurs during successive years. A value of 2.3% yr^-1^ represents the percentage of forest floor covered by mounds at the maximum annual rate of mound formation measured in a 0.5–ha plot in this study (712 mounds ha^-1^ yr^-1^). We used predictions from the no disturbance simulation for litter layer mass and N and P content of portions of the forest floor that were not buried by pocket gopher mounds, and used predictions from the single mound simulation for locations covered by pocket gopher mounds. The balance between these two simulations were weighted as a proportion of one of the five simulated rates of new mound formation. We tracked predicted values for newly vs. previously formed mounds in a matrix that updated mass of each annual cohort of the four types of litter (buried pine, buried oak, surface pine and surface oak) every year in the Microsoft Excel spreadsheet model. For N and P contents of annual litter cohorts, we used the relationship shown in Fig 4 and S3 Table to estimate N concentrations using Equation 2, and a similar function for P concentrations. As in the previous simulations, we simulated Phase 1 of the decomposition process, and annual litter cohorts that had less than 20% mass remaining were excluded from integrated values. Values from the four types of annual litter cohorts were summed for each year. We then integrated results from each time step to calculate the net effect of mound density on litter layer mass and N and P content.

**Prescribed fire simulations**

For low-intensity prescribed burn simulations, we initially assumed that litter layer mass was in “steady state”, as predicted in the no disturbance simulations. We used an estimated value of 50% consumption of annual litter cohorts during Phase 1 of decomposition during each fire, based on published values for low-intensity fires in longleaf pine forests [53,54] and mixed southern pine forests [55]. We simulated consumption of the litter layer by prescribed fires at 3, 5 and 10–year intervals, based on typical fire return intervals employed at Ordway Swisher Biological Station. We used Equation 2 and the relationships in Figure 4 and S3 Table to estimate the N and P content of litter, and Equation 3 to estimate the amount of forest floor remaining following each prescribed fire. We assumed that N was volatilized and P was pyro-mineralized in the same proportion as consumed litter. We further assumed that the litter remaining in annual litter cohorts that was not consumed decomposed as surface litter in the litterbag study (i.e., prescribed burning had no effect on rates of litter decomposition), thus net accumulation of litter mass and N and P in litter was assumed to follow the no disturbance simulations. Between prescribed fires, we assumed that annual canopy litterfall mass, composition and nutrient content was unaffected by low intensity burns. Values from the two types of annual litter cohorts (surface pine and surface oak) were summed for each year simulated. We then integrated results from each time step to calculate the net effect of prescribed fires on litter layer mass and N and P content in litter.

**Mound density and fire simulations**

To evaluate how the density of pocket gopher mounds affects litter layer consumption during prescribed fires, we used the simulation model to explore the interactive effects of variation in the rates of pocket gopher mound formation and prescribed burn intervals on litter layer mass and N and P dynamics of the litter layer. As in the mound density simulations, five densities of mounds were simulated, covering no (0% yr^-1^), low (1% yr^-1^), medium-low (2.3% yr^-1^), medium-high (5% yr^-1^), and high (10% yr^-1^) area of the forest floor per year. Simulated return intervals for prescribed burns followed the prescribed fire simulations, and 3, 5 and 10–year return intervals were simulated. Annual cohorts of litter were simulated as in the mound density simulations for newly formed mounds, and as prescribed burn simulations for areas without pocket gopher mounds. For older mounds that burned, we assumed that average annual litterfall amounts accumulated on mounds through time, and decomposition and N and P dynamics were modeled as in the no disturbance simulations until prescribed burns occurred. We also assumed that reduced litter amounts on newer mounds had no effect on the percent consumption (50% of initial litter amount) during prescribed burns. Annual cohorts were summed each year, and values are presented as percent reduction in litter layer consumption, N volatilization and P pyro-mineralization compared to predictions of the prescribed fire simulations in the absence of pocket gopher mounds.

**References**

76. Harrington TB, Edwards MB. Understory vegetation, resource availability, and litterfall responses to pine thinning and woody vegetation control in longleaf pine plantations. Can J For Res. 1999;29: 1055–1064.

77. Brockway DG, W. Outcalt K. Gap-phase regeneration in longleaf pine wiregrass ecosystems. For Ecol Manage. 1998;106: 125–139. doi:10.1016/S0378-1127(97)00308-3

78. Samuelson LJ, Stokes TA, Butnor JR, Johnsen KH, Gonzalez-Benecke CA, Martin TA, et al. Ecosystem carbon density and allocation across a chronosequence of longleaf pine forests: Ecol Appl. 2017;27: 244–259. doi:10.1002/eap.1439

79. Binkley D, Richter D, David MB, Caldwell B. Soil chemistry in a loblolly/longleaf pine forest with interval burning. Ecol Appl. 1992;2: 157–164. Available: http://www.jstor.org/stable/1941772

80. Clark KL, Gholz HL, Castro MS. Carbon dynamics along a chronosequence of slash pine plantations in north Florida. Ecol Appl. 2004;14: 1154–1171.

81. Powell TL, Gholz HL, Clark KL, Starr G, Cropper WP, Martin TA. Carbon exchange of a mature, naturally regenerated pine forest in north Florida. Glob Chang Biol. 2008;14: 2523–2538. doi:10.1111/j.1365-2486.2008.01675.x
